# Role of Lake Aquatic–Terrestrial Ecotones in the Ecological Restoration of Eutrophic Water Bodies

**DOI:** 10.3390/toxics11070560

**Published:** 2023-06-26

**Authors:** Tingting Dai, Rui Liu, Xingxing Zhou, Jing Zhang, Mengting Song, Ping Zou, Xiaoyi Bi, Shuibing Li

**Affiliations:** 1 Institute of International Rivers and Eco-Security, Yunnan University, Kunming 650091, China; daitt528@mail.ynu.edu.cn; 2 School of Ecology and Environmental Science, Institute for Ecological Research and Pollution Control of Plateau Lakes, Yunnan University, Kunming 650091, China; zouping0529@outlook.com (P.Z.); bixiaoyi@126.com (X.B.); nutritionhealth@163.com (S.L.); 3 Architecture and Environment, Ningxia Institute of Science and Technology, Shizuishan 753000, China; zxingxing1209@163.com; 4 International School of Shenyang Jianzhu University, Shenyang 110168, China; lemon_tea777@163.com; 5 School of Resources and Materials, Northeastern University at Qinhuangdao, Qinhuangdao 066064, China; song1085259335@163.com

**Keywords:** eutrophication, lake aquatic–terrestrial ecotones, bioremediation, plant community, in situ treatment

## Abstract

Freshwater lake eutrophication is a global concern causing adverse effects on aquatic ecosystems. The degradation of lake aquatic–terrestrial ecotones, which are the transitional zones between terrestrial and water ecosystems, contributes to eutrophication. These ecotones play vital roles in nutrient cycling, runoff control, biodiversity conservation, and habitat provision. In the past three decades, the research on lake aquatic–terrestrial ecotones has focused on techniques for managing contaminants and runoff purification. This paper reviews the recent studies on the restoration ability of eutrophic water bodies in lake aquatic–terrestrial ecotones in recent years regarding three aspects: the establishment, restoration mechanism, and improvement of restoration function. In addition, ecological factors such as lakeshore height, water level, surface runoff, shallow groundwater level, and rainfall intensity have impacts on the restoration capacity of lake aquatic–terrestrial ecotones.

## 1. Introduction

The deterioration of lake water environments, especially the increase in freshwater lake eutrophication, is prevalent worldwide [1]. Eutrophication drives a series of adverse effects such as algal blooms, aquatic vegetation decline, fish mortality, and eventual ecosystem degradation and collapse [2]. One main cause of eutrophication is the degradation of lake aquatic–terrestrial ecotones and the loss of their ecological functions [3]. Aquatic–terrestrial ecotones are areas between terrestrial ecosystems and water ecosystems [4]. They play crucial roles in hydrological, biological, and geochemical cycles linking terrestrial and aquatic ecosystems, including the transport and transformation of nutrients (including nitrogen and phosphorus) [5], runoff control, microclimate regulation, and biodiversity protection [6]. Ecotones protect lake ecosystems by reducing pollutant inputs, and they are an indispensable organic component of a healthy lake ecosystem [7]. Lake aquatic–terrestrial ecotones can be both a sink and source of nutrients [8], as well as a species source (gene pool) and important habitat for lake wildlife [9]. A special property of lake aquatic–terrestrial ecotones includes seasonal variations in water levels that create anaerobic conditions for flora and fauna that are essential to these zones [10]. The characteristics and special status of lake aquatic–terrestrial ecotones in watershed ecosystems with low operational and maintenance costs have recently received attention from international ecological and environmental communities [11]. 

**Figure 1:**
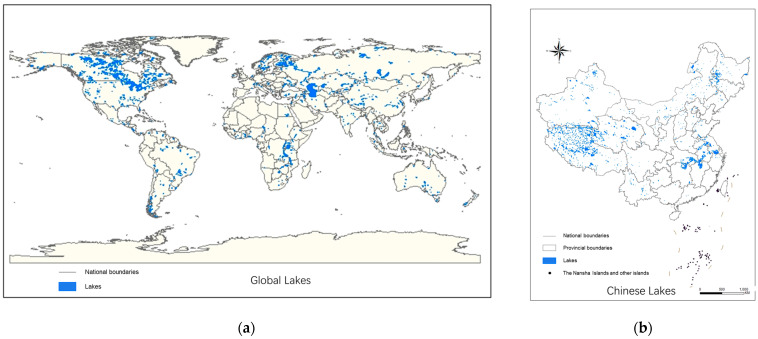
Lake distribution map: (**a**) worldwide; (**b**) China. The data come from the National Earth System Science Data Center of the National Science and Technology Infrastructure of China.

There are many lakes in the world, and most freshwater lakes experience varying degrees of eutrophication [12,13,14]. Detailed information on the distribution of lakes across the world and China is shown in Figure 1. Freshwater lake ecosystems are not only valuable human resources but also crucial environmental systems [15]. Therefore, studying the ecological restoration effects of lake aquatic–terrestrial ecotones on eutrophication is of great significance. Chinese research on lake aquatic–terrestrial ecotones began in the early 21st century [16], mainly focusing on freshwater lakes such as Erhai Lake, Taihu Lake, Dianchi Lake, and the Three Gorges Reservoir [17,18,19,20]. This research led to the development of practical techniques such as low-biomass grass planting, buffer zone protection, and surface runoff purification. Similar research on lake aquatic–terrestrial ecotones in America, Europe, and Japan has focused on controlling contaminants in agricultural runoff [21,22,23]. That is why some of the research has focused on streams and rivers, which are far more numerous than lakes, and because most people live along them, they face more pollution from agricultural nonpoint sources than lakes. In recent years, foreign research has focused more on the maintenance and management of lake aquatic–terrestrial ecotones [24]. Nsenga et al. [25] found that extreme weather can affect the design implementation process of lake aquatic–terrestrial ecotones, and cold weather possesses the most detrimental impact. Rinku et al. [26] proposed an integrated approach to managing lake aquatic–terrestrial ecotones. The knowledge framework of lake aquatic–terrestrial ecotones has expanded in recent years. However, progress at the global scale has been limited, mainly due to the extensive functional and structural diversity of these zones. Improving our understanding requires the reasonable characterization of the extent of lake aquatic–terrestrial ecotones based on multiple factors such as lake type, width, rainfall, vegetation type, soil properties, slope, and adjacent land use. Through a thorough analytical exploration of the literature, this paper explores the ecological restoration potential of lake aquatic–terrestrial ecotones and provides insights into their management and conservation in combating freshwater lake eutrophication.

## 2. Bioremediation of Lake Aquatic–Terrestrial Ecotones

### 2.1. Design and Application of Lake Aquatic–Terrestrial Ecotones

The effectiveness of aquatic–terrestrial ecotones in reducing nonpoint source pollution has been recognized and utilized for a long time [27,28,29,30]. When establishing a lake aquatic–terrestrial ecotone, a wide lake aquatic–terrestrial ecotone with a reasonable vegetation density is considered a “natural barrier” for preventing nonpoint source pollution in a lake. However, due to land resources and geographical constraints, it is not always possible to grow the most effective vegetation types or indiscriminately expand the width of a lake ecotone to improve its effectiveness in preventing nonpoint source pollution. According to a relevant retrieval from the Web of Science, there are mainly four methods for determining the widths of lake ecotones, including shallow-water lakes, deep-water lakes, and freshwater lakes. Detailed information is shown in Table 1. Borin et al. [31] found that nitrate-nitrogen in surface runoff decreased from 50% to 20% when the broadband of a lakeshore intersection zone was reduced from 16 to 8 m. Haycock et al. [32] identified that the most nitrate is removed within the first 8 m of entering the riparian zone, with overall interception rates of greater than 80% for sediment and 50% for total phosphorus in buffer zones of greater than 10 m.

Plants are the main biological component of lake ecotones. The use of vegetated buffer zones to control external nutrient inputs is key to mitigating eutrophication [38]. The riparian vegetation composition impacts the overall plant community [39]. Plants adsorb, enrich, and degrade pollutants through their roots, stems, and leaves. Plants rely on their metabolism combined with the action of microorganisms to absorb large amounts of harmful substances such as nitrogen, phosphorus, and suspended matter in eutrophic water bodies [40]. Plant communities involve various plant species and create habitats for organisms, and the vegetation in lake ecotones can effectively increase the phosphorus content in sediments, gradually migrating the phosphorus fraction to favor plant growth and uptake [41]. Vegetation increases the phosphorus storage capacity of sediments in the interlaced zones of rivers and lake banks. Increased riparian vegetation improves ecological functions such as nutrient sorption, sedimentation, and retention in the interlaced zones, further reducing nutrient inputs from land to water bodies to improve lake water quality [42].

When establishing powerful plant communities, aquatic plants are key to repairing eutrophication. The deep-water area to the shoreline encompasses various vegetation zones, including the submerged vegetation zone, floating-leaf vegetation zone, floating vegetation zone, and emergent vegetation zone, and involves a multi-seasonal composite structure of aquatic plant communities. Constructing four levels of vegetation zones enables land-based pollutants to be effectively filtered and retained, purifies water quality, and improves water transparency. Over time, nitrogen and phosphorus are removed from eutrophic water bodies by harvesting the above-ground biomass [43]. Plant species significantly influence the ability of lake ecotones to remediate nutrient loads [44]. As shown in Table 2, different plants have different removal rates for high levels of nitrogen and phosphorus in eutrophic water bodies. Aquatic plant selection should initially focus on common species suitable for the local geography, climate, and conditions with a reasonable planting density to form a diverse plant community [19]. One study demonstrated that the long-term soaking of vegetation releases large amounts of dissolved organic matter (DOM) into the water [45]. This provides nutrients for bloom-forming species, which may promote the rapid reproduction of these algae, instigating blooms [45]. Consequently, exploring the effects of more DOM sources on algal growth could significantly enhance capacities for bloom control [46]. 

The most effective riparian vegetation buffer zone pattern includes terrestrial areas that combine tree infestation–grass patterns. Hu et al. [52] found that buffer strips with herbaceous plants were effective in retaining surface water and pollution runoff, with a removal rate of over 30% for both runoff pollutants and retained surface seepage pollutants. Yan et al. [53] identified that natural vegetation is more effective than artificial vegetation in absorbing pollutants, which is probably due to natural selection and local adaptation. Terrestrial mixed forests with grasses are better at absorbing runoff pollutants than woodland or grassland in isolation. Poeppl et al. [54] revealed that a mixture of wetland and forest cover with natural vegetation planted in a buffer strip significantly reduced runoff sediment inputs into the water body. Forested riparian buffer strips with well-developed root systems can absorb more nutrients, remove nitrate more effectively, and stabilize stream banks, thereby reducing streambank erosion. A more complex plant community structure results in an improved ecological buffer function in a lake ecotone.

Numerous factors such as plant biomass, temperature, light conditions, plant type, purification time, water velocity, and the physicochemical properties of the water can affect plant efficiency in removing pollutants. The higher the plant biomass [55], the more nutrients can be removed. Longer root systems can increase the favorable conditions for the microbial decomposition of organic matter. Aquatic plant selection incorporating some strong aquatic plants with developed root systems can improve the slope fixation capacity. Increased concentrations of allelochemicals released by plants into the water can also increase algae suppression over time. Screening for the optimal vegetation suited to local conditions is particularly important in the ecological restoration of eutrophic water bodies. Therefore, the type of ecotone vegetation cover is important, and the sediment and hydrology of the buffer zone warrant consideration. Additionally, plant selection should screen for flood- and drought-tolerant species. One plant species can have different nutrient removal effects under different environmental conditions. Therefore, relying on a single plant may not achieve improved degradation of all types of nutrients in all conditions [56], so plant combinations are needed. However, special attention should be paid to the growth characteristics of plants and their affinity and aggressiveness with other species in plant configurations. Fast-growing species should be propagated in appropriate proportions to restrict conflicts with slower-growing species. Slower-growing species may require planting in larger proportions to develop a successful plant mix. Plant selection and configuration should be considered on a site-specific basis (e.g., following the current riparian structure and river water quality) to sustainably enhance new ecosystems in riparian zones [57]. Currently, little information is available on riparian vegetation characteristics (composition, density, and layout). Understanding riparian vegetation requires specialized research to optimize plant configurations and identify locally appropriate riparian vegetation configuration patterns for sustained pollution control.

### 2.2. Bioremediation Mechanisms

A lake ecotone’s vegetation buffer zone has a minimum width requirement to effectively control or reduce pollutants and achieve water purification. The main action mechanisms include the retention of pollutants in runoff, absorption by vegetation, adsorption by soil, and degradation of pollutants by microorganisms [58]. Furthermore, the agglomerated structure of a lake ecotone increases the mechanical strength and impact resistance of the inter-rooted soil, allowing soil consolidation and slope protection [59]. Plants in lake ecotones promote nutrient uptake, deposition, and infiltration by altering the runoff velocity and increasing the hydraulic residence time [60]. When plants reach saturation with adsorbed nutrients, the phosphorus sorption capacity in lake ecotones decreases with an increasing hydraulic residence time. Plant roots can promote runoff filtration. When runoff-carrying pollutants pass through the vegetation buffer zone, the dissolved pollutants enter the soil with the infiltrated water and are absorbed by the plant roots. Moreover, a more developed root system from a high biomass of vegetation in a lake ecotone enables increased plant root uptake and microbial degradation, thus improving the retention efficiency of pollutants in runoff [61]. The roots of many plants can secrete a variety of organic compounds. This is especially evident in phosphorus-deficient conditions, wherein aquatic plants continuously secrete large amounts of low-molecular-weight organic compounds into the growth medium, providing large amounts of nutrients and energetic substances for inter-rooted microorganisms and altering microbial activity, biomass, and ecological distributions. 

Microbial degradation plays a crucial role in the removal of nitrogen and phosphorus in lake ecotones. As the decomposers in ecosystems, microorganisms contribute significantly to pollutant removal and nutrient recycling. In high-nutrient ecosystems, microorganisms particularly influence nitrogen dynamics because plants cannot be directly absorbed and used by plants. Soil microorganisms convert organic nitrogen into inorganic nitrogen through processes such as ammonification, nitrification, and denitrification, thereby promoting the biogeochemical nitrogen cycle in water bodies. Phosphorus removal in lake ecotones primarily occurs through soil particle deposition, sorption, and plant uptake of dissolved and particulate phosphorus. Particle sedimentation is a significant process in the removal of particulate phosphorus during surface runoff, overland flow, and floodplain inundation, especially when the runoff carries a high concentration of dissolved phosphorus into lake ecotones [62]. Soluble phosphorus enters a lake through various pathways, including microbial assimilation and uptake, soil humus uptake, and infiltration into groundwater. Additionally, solid phosphorus can be deposited in lake ecotones through sedimentation when mixed with other suspended solid particles in surface runoff [63]. Plant roots and leaf surfaces can also intercept and capture solid phosphorus through adsorption from surface runoff. Furthermore, microorganisms contribute to the decomposition of organic phosphorus, providing nutrients for plant growth and development.

### 2.3. Improvement of Ecological Restoration Effect and Management of Lake Ecotones

Lake ecotones are rich in microbial species, including bacteria, mycobiota, actinomycete, protozoa, and metazoans. Among them, bacteria are one of the most abundant and complex taxa. The number of bacteria per gram of soil can be in the hundreds of millions or even billions. Microorganisms are usually adsorbed on solid surfaces, including those of plants [64]. Plants in lake ecotones can remove nitrogen and phosphorus through direct absorption and indirectly through increased microbial activity by altering the redox potential within the wetland. Kickuth’s root zone theory states that plants in lake ecotones can transport the oxygen produced via photosynthesis to the root zone through sparse tissue, so that an aerobic zone is formed near the root zone, with areas farther away from the root zone becoming anoxic, and areas that are even further becoming anaerobic [65]. Therefore, bacteria in this zone can include aerobic bacteria, anaerobic bacteria, facultative anaerobic bacteria, and other species. There are distinct aerobic and anaerobic bacterial communities in a lake ecotone. Moreover, the local microbial population is the result of a combination of dissolved oxygen content, chemicals released by plants, and soil properties. Annually, the microbial population in a lake ecotone is low in spring, starts to increase in summer, reaches a maximum in autumn, and then decreases in winter. In this population, facultative anaerobic bacteria (which can survive in both anaerobic and aerobic conditions) are the optimal species for pollutant degradation. The facultative anaerobic bacteria isolated by Smirnova et al. had a high degradation efficiency for cellulose in plants and produced limited nitrogen fixation [66].

Pollutants serve as carbon and energy sources for the growth and reproduction of microorganisms or produce intermediate metabolites that provide essential nutrients, thereby stimulating the secretion of active enzymes for pollutant degradation [67]. During nitrogen and phosphorus removal, microorganisms control the efficiency of nitrogen pollutant mitigation by directly participating in the mineralization and ammonia oxidation of organic matter and driving ecosystem services in vegetated buffer ecosystems. Recent findings suggest that plant root secretions can stimulate plant-associated microorganisms, thereby enhancing the biodegradation of pollutants in lake ecotones [68]. Inter-rhizosphere microorganisms are the most sensitive soil microbial community to plant community changes and are also the nutrient regulator between soil and root systems. Inter-rhizosphere bacteria in soil play key roles in plant growth, development, and environmental adaptation. Studies on nitrate removal in wetlands and riparian zones have demonstrated that rhizosphere bacterial metabolism is linked to the removal of these inorganic nutrients, primarily through denitrification [69]. Therefore, studying the diversity of inter-root soil bacterial communities and their functions deepens our understanding of the relationships between plants and soil microorganisms and provides guidance for the bioremediation of degraded ecosystems [70].

Microorganisms are the main carriers and bearers of nitrogen- and phosphorus-containing pollutants and organic pollutants in eutrophic water bodies. Enhancing the performance and function of microorganisms involved in nitrogen and phosphorus removal in lake ecotones is crucial for ecological restoration. To enhance the capacity of microorganisms in the lake ecotones of eutrophic water bodies, strategies could include introducing new bacteria, leveraging the diverse physiological characteristics of different microorganisms, systematically screening for optimal bacteria, combining multiple microbial strains into a composite microbial treatment, and employing suitable bacterium encapsulation technology (to enhance their overall ecological effectiveness). Wu et al. [71] found that chlorophyll a, total nitrogen, total phosphorus, and the permanganate index in the water column decreased after inputting effective microbial flora, while the dissolved oxygen content and transparency of the water column increased accordingly. Chen et al. [72] used microbial eco-remediators in a eutrophic artificial landscape lake, causing the water column chlorophyll a (Chla), COD, and TP concentrations to decrease by 73.03%, 50.62%, and 65.48%, respectively. Gao et al. [73] identified the highest degradation rates of micro-ecological agents for COD_Cr_, TP, and TN as 33.57%, 83.33%, and 42.98% in the overlying water, respectively, and 31.16% for TN and 19.53% for organic carbon in the substrate. It can be seen that adding microbial communities is a crucial step in establishing a microbial augmentation system. In addition, if indigenous microbial strains can be screened from eutrophic water bodies, they will have better remediation effects compared with commercial strains. Indigenous microbial communities have a greater capacity for adaptation to lake environments, enabling faster acclimatization [74]. Even the addition of some effective microbial communities can increase dissolved oxygen in water bodies [75]. 

Nevertheless, the practical applications of adding microbial communities to enhance microbial systems in eutrophic water bodies face certain limitations. These include the susceptibility of microorganisms to washout by flowing water, their short action durations, and the challenge of stabilizing treated water due to predation by other organisms in stagnant water. Furthermore, the lack of carriers for adsorption, growth, and reproduction makes their preservation and transport difficult [76]. Fortunately, the emergence and advancement of microbial immobilization treatment technology have effectively addressed these challenges. Currently, microbial immobilization technology holds promise for the remediation of eutrophic water bodies. Immobilized microorganisms exhibit effective inhibition of algae proliferation, thereby contributing to water purification. Encapsulation is the most extensively studied immobilization method [77]. Among various materials, studies have demonstrated the effectiveness of sodium alginate in immobilizing bacteria [78,79,80]. Further research should investigate the mechanisms of mutual influence between microbial communities and their interactions across various aspects including microbial growth and metabolism. Additionally, research is needed to assist in screening high-quality strain combinations and exploring the best mixing ratio to stimulate the maximum advantage of each microbial community to achieve ideal degradation and pollutant removal. 

In the management of lake ecotones, implementing an optimal plant harvesting program is equally important for the long-term control of pollutants in the lake ecotones [81]. Nutrients and other pollutants absorbed by plants are released into the water when plants die and decay, especially over winter. Conversely, fallen leaves and wood can serve as organic matter, an essential source of carbon (energy) for the river food web to facilitate the removal of pollutants. In addition, periodic selective harvesting may encourage the growth of smaller plants that may absorb nutrients more rapidly than mature plants. Therefore, we must assess the appropriate trade-offs between the risks and benefits of plant harvesting. Further research into and development of appropriate plant-harvesting strategies is critical for continued contaminant control [82].

## 3. Influences of Ecological Factors on the Role of Lake Aquatic–Terrestrial Ecotones

### 3.1. Lakeshore Height

The height of the lakeshore can influence the lakeshore moisture conditions. Many riparian ecosystem functions, such as chemical reduction, are related to soil moisture. The soils on high lakeshores (h ≥ 1 m) are influenced by the distance from the water table. The impact on biogeochemical processes, such as denitrification in the topsoil, may be reduced with low soil moisture. Conversely, riparian soils with high riparian vegetation and low soil moisture can act as surface runoff sinks. However, in the riparian soil on a low lakeshore (h ≤ 0.3 m), short vegetation very close to the water table can become very shallow seepage zones. This can lead to high moisture conditions with saturated terrestrial flows, reducing the biogeochemical processing of many elements.

The soil matrix potential in medium–high lake aquatic–terrestrial ecotones is more variable than in low lake aquatic–terrestrial ecotones due to greater changes in groundwater levels. However, a more accurate prediction of soil moisture conditions relies on correlating riparian height with detailed field descriptions, including various local factors such as soil type. Lakeshore height can be easily assessed in the field, ensuring its place as one of the riparian characteristics that environmental managers include when predicting the potential of lake aquatic–terrestrial ecosystem services [83].

### 3.2. Water Levels

Lake aquatic–terrestrial ecotones are characterized as both terrestrial and aquatic ecosystems. Annually, plants growing in these areas may be completely submerged in water at some stage during the growing season, but they can be exposed to drought stress at other times. Changes in water level can lead to changes in redox conditions in lake ecotones, and they can also exert some stress on the growth of plants. Moreover, when there is a significant difference in the lake water level, there are significant differences in the soil microbial communities in vegetation buffer zones [84,85,86]. At high water levels (a high water level for a deep lake is 40–50 m, and a high water level for a shallow lake is 10–20 m), the soil redox potential is the main factor affecting the soil microbial community composition in the vegetated buffer zone. At low water levels (a low water level for a deep lake is 20–30 m, and a low water level for a shallow lake is <10 m), total nitrogen better explains the changes in the soil microbial community composition [84]. Zhang et al. [87] suggested that the wet bacterial network is more stable and complex than the dry bacterial network. In summer, due to the high temperatures during this time, the vegetation in a lakeside ecotone quickly recovers and reaches its growth peak at the end of summer [88,89], absorbing and fixing large amounts of nutrients from the soil and water. When a portion (or even all) of the vegetation in lake ecotones is flooded, the vegetation is subjected to prolonged inundation stress, causing leaves to fall and decompose and potentially releasing large amounts of organic matter and nutrients into the water column [90]. As an endogenous factor, inundation may increase nutrients in the reservoir, becoming a pollution source and deteriorating water quality. However, at high water levels, the denitrification rate increases with the intensity of the inundation. This is because the denitrification rate doubles and the efficiency of nitrogen removal by plants increases with periodic flooding compared with non-flooding due to the denitrification process favoring anoxic/anaerobic conditions [91].

There is a close relationship between water levels and carbon emissions in lake ecotones. Carbon is absorbed by vegetation during the growing season and released into the surrounding environment during the high-water-level season. At low elevations, GHG emissions are greater in lake aquatic–terrestrial ecotones than in permanently flooded areas, and at high elevations, GHG emissions are greater in permanently exposed areas than in permanently flooded areas [92]. The wet/dry cycle affects nutrient release by altering both the sediment properties (including water content and porosity) and the oxygen conditions in the riparian zone, influencing greenhouse gas production [93]. To mitigate the contribution of vegetation residues to GHG emissions, appropriate vegetation regulation and management strategies (such as harvesting, grazing, and reusing harvested biomass as potential fertilizer) should be implemented prior to flooding to reduce carbon emissions from riparian zones [94].

### 3.3. Surface Runoff

Lake ecotone management of sediment and pollutants for surface runoff is crucial, particularly in areas with significant soil erosion within the watershed [95]. However, the effectiveness of lake ecotones in retaining sediments and pollutants is influenced by the runoff intensity and flow velocity. A higher rainfall intensity and runoff volume result in increased kinetic energy, which can limit the capacity of an ecotone to manage sediments and particulate pollutants. Similarly, higher runoff velocities hinder the interaction between pollutants and vegetation in ecotones, reducing the efficiency of pollutant retention [96]. Moreover, the pattern of the runoff flow also plays a key role in an ecotone’s ability to manage sediments and pollution. Concentrated runoff patterns enhance the vegetation–soil–microbe interactions in the buffer zone system, thereby improving the retention efficiency [97]. Concentrated flow through lake ecotones leads to significantly higher pollutant removal rates compared with uniform flow. This is due to the intensified vegetation–soil–microbe interactions in the buffer zone systems, which enhance pollutant retention [97]. The presence of vegetation in lake ecotones increases soil roughness, effectively reducing the runoff velocity and thereby diminishing the transport capacity of the surface runoff containing pollutants, such as nitrogen and phosphorus. This reduction facilitates the deposition of coarse particles that are predominantly transported in large masses.

### 3.4. Shallow Groundwater Level

A seasonal shallow water table surrounds an aquatic–terrestrial ecosystem. Carluer et al. [98] revealed that the shallow water table (<1.8 m) is an important factor affecting the performance of a lake aquatic–terrestrial ecotone. The direction and location of the groundwater flow can impact the reduction efficiency of the lakeshore intersection zone, which is related to the soil saturation phenomenon caused by the shallow groundwater table [99]. Groundwater levels rise and fall in the soil’s internal water-saturated layer and non-water-saturated layer. The two parts of the soil layer work closely together, affecting the internal structure of the soil, soil moisture, and microbial community succession patterns, resulting in changes in the water and solutes in the soil layer. Conversely, in engineering, soil stress mainly includes effective stress and void water pressure, which can be transformed into each other under certain conditions. Therefore, changes in groundwater levels lead to changes in soil stress and void water pressure, which lead to soil deformation. The soil deformation precedes changes in the soil permeability coefficient, further affecting the transport of pollutants. Lauvernet et al. [100] identified that the rise and fall in water tables alter the importance of different factors (the length of lake ecotones, soil properties, etc.) that affect the vegetation performance of lake ecotones over a range of depths. In the absence of a water table, the surface runoff reduction efficiency is highly sensitive to saturated soil hydraulic conductivity. Conversely, when a shallow water table is present, the effect of saturated soil hydraulic conductivity on surface runoff decreases as the influence of the water table depth increases.

### 3.5. Rainfall Intensity

Rainfall is important in generating surface source pollution. Rainfall generates runoff that scours and leaches pollutants from the land surface and carries them into water bodies, causing pollution. When the rainfall intensity exceeds the surface infiltration intensity, surface runoff is generated. The intensity and temporal characteristics of rainfall determine the temporal characteristics of surface source pollution [101]. Konapala et al. [102] showed that in areas with high rainfall seasonality, seasonal rainfall will be more erratic in the future. Conversely, areas with lower rainfall seasonality will experience increased rainfall during the rainy season. Shen et al. [103] showed that frequent rainfall increased soil water content, which in turn affected the sediment and pollution retention of lake ecotones.

## 4. Conclusions and Prospects

Since many lakes suffer from eutrophication worldwide, the potential of lake aquatic–terrestrial ecotones needs to be explored and used for maintaining and improving lake water quality. We can also learn from the research conducted on river riparian zones in order to select transferable knowledge and identify the limitations of the effects of lake aquatic–terrestrial ecotones. Furthermore, in response to the characteristics of slow water exchange and easy diffusion of pollutants in lakes, aquatic–terrestrial ecotones suitable for lakes could be designed. Future research could be dedicated to the following areas:

(1) Understanding ecological dynamics: Conducting in-depth studies to comprehend the ecological dynamics of lakeside ecotones, including nutrient cycling, pollutant diffusion, and species interactions. This understanding will inform the development of targeted management strategies.
(2) Ecosystem restoration techniques: Investigating and developing effective techniques for restoring and enhancing lakeside ecotones. This involves exploring vegetation patterns, buffer zone designs, and restoration methods that maximize pollutant removal and enhance ecological resilience.
(3) Integrated management approaches: Promoting the adoption of integrated management approaches that consider the interconnectedness of various factors affecting lakeside ecotones, such as water quality, sedimentation, and nutrient inputs. This holistic approach will facilitate more comprehensive and efficient ecosystem management.
(4) Monitoring and assessment: Implementing robust monitoring and assessment programs to evaluate the effectiveness of management strategies and track the ecological health of lakeside ecotones over time. This data-driven approach will enable adaptive management practices and continual improvement.

## Figures and Tables

**Table 1:** Methods for determining the widths of lake ecotones.

Method	Applicable Scope	References
A hydrographic aquatic–terrestrial ecotones model	Deep-water lakes	[33,34]
Ratio method: the maximum ratio of environmental benefits obtained from land structure adjustment around lakes to investment funds		
	Freshwater lakes	[35]
Numerical simulation method: using waves with numerical simulation	Large shallow lakes	[36]
Determining the width of a lake ecotone through nutrient removal rate (TN, TP)	Freshwater lakes	[37]

**Table 2:** Detailed information on removal rates of different types of plants.

Plant Species	Removal Rate/%	Reference
*Vallisneria natans*	TN-81.2/TP-90.8	
*Potamogeton distinctus*	TN-86.6/TP-86.2	[47]
*Hydrilla verticillata*	TN-75.6/TP-81.3	
*Eichhornia crassipes*	TN-42.44/TP-96.44	[48]
*Pandanus*	NO_3_^−^N-100/PO_4_^3−^P-64	[49]
*Nelumbonucifera*	TN-76.87/TP-76.47	[50]
*Phragmites australis*	TN-69	[51]

## Data Availability

The data presented in this study are available upon request from the corresponding author.
